# Supplementary material for: Echinococcus multilocularis Calreticulin Inhibits Lectin Pathway of Complement Activation by Directly Binding to Mannose-Binding Lectin
Source: Pathogens. 2025 Apr 5;14(4):354. doi: 10.3390/pathogens14040354 (PMC12030537; doi:10.3390/pathogens14040354)
Supplement: Supplementary file 1 [file pathogens-14-00354-s001.zip › Supplementary/Supplementary.pdf]

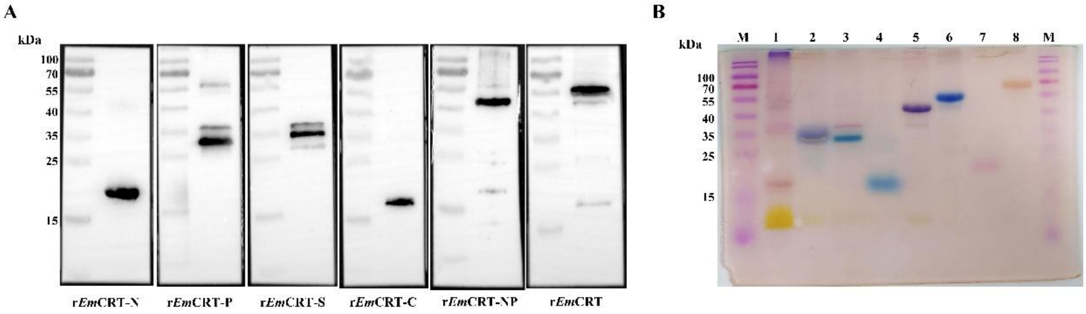

Supplementary Figure S1. (A) r*Em*CRT and its fragments recognized by anti-His mAb; (B) Identification of r*Em*CRT and its fragmentation by Stains-all. M: protein marker, 1: *Em*CRT-N, 2: *Em*CRT-P, 3: *Em*CRT-S, 4: *Em*CRT-C, 5: *Em*CRT-NP, 6: *Em*CRT, 7: *Em*Fer, 8: BSA.

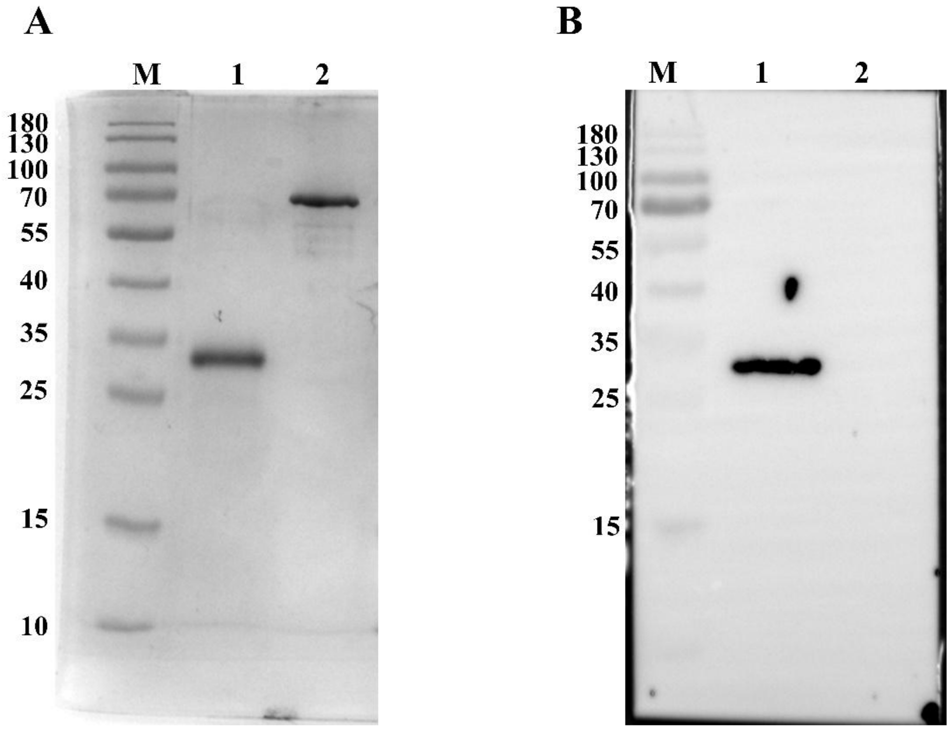

Supplementary Figure S2. (A) SDS-PAGE of MBL and BSA. M: protein marker, 1: MBL (1 μg), 2: BSA (1 μg); (B) r*Em*CRT binding to human MBL by Far western blot. M: protein marker, 1: MBL (1 μg), 2: BSA (1 μg).

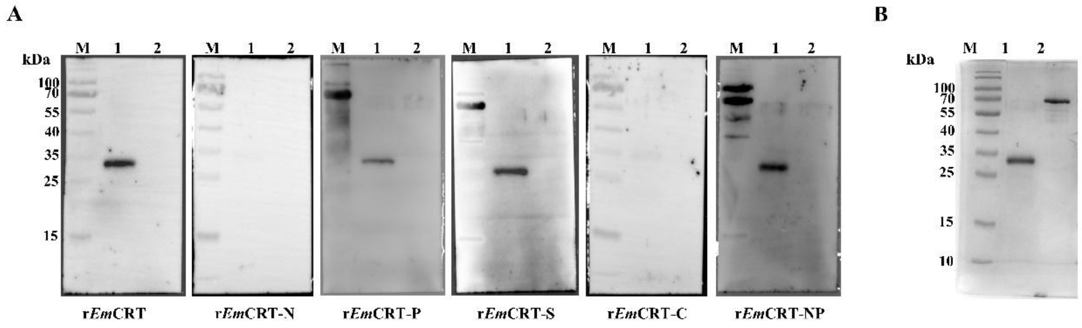

Supplementary Figure S3. (A) r*Em*CRT and its fragments binding to human MBL by Far western blot. M: protein marker, 1: MBL (1 μg), 2: BSA (1 μg); (B) SDS-PAGE of MBL and BSA. M: protein marker, 1: MBL (1 μg), 2: BSA (1 μg).
