# Supplementary figures and images for: Echinococcus multilocularis Calreticulin Inhibits Lectin Pathway of Complement Activation by Directly Binding to Mannose-Binding Lectin
Source: Pathogens. 2025 Apr 5;14(4):354. doi: 10.3390/pathogens14040354 (PMC12030537; doi:10.3390/pathogens14040354)

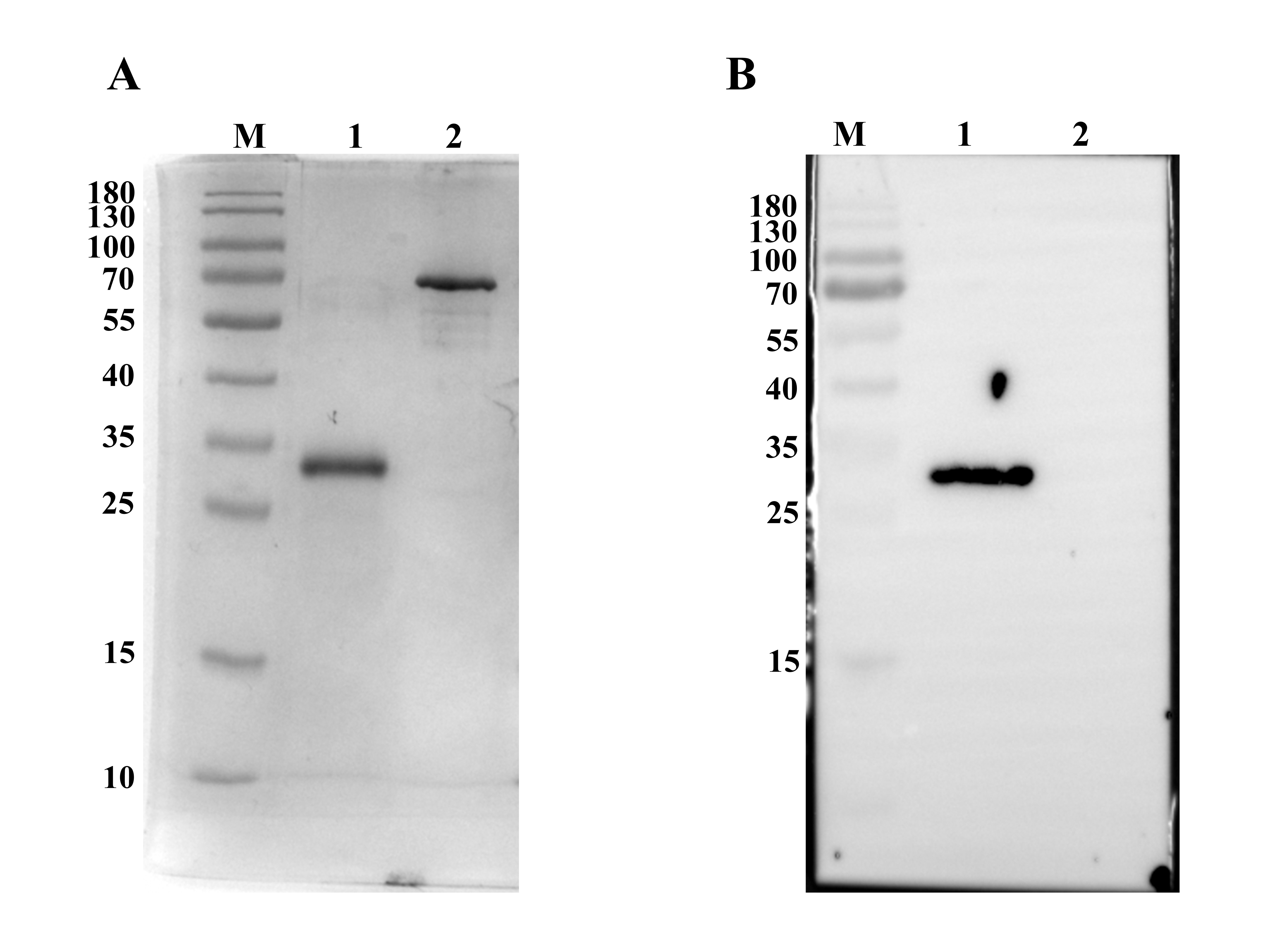

Supplement: Supplementary file 1 [file pathogens-14-00354-s001.zip › Supplementary/Figure S2.jpg]
